# Association Between Infant Mortality Attributable to Birth Defects and Payment Source for Delivery — United States, 2011–2013

**DOI:** 10.15585/mmwr.mm6603a4

**Published:** 2017-01-27

**Authors:** Lynn M. Almli, Caroline C. Alter, Rebecca B. Russell, Sarah C. Tinker, Penelope P. Howards, Janet Cragan, Emily Petersen, Gerard E. Carrino, Jennita Reefhuis

**Affiliations:** ^1^Division of Congenital and Developmental Disorders, National Center on Birth Defects and Developmental Disabilities, CDC; ^2^March of Dimes Foundation, White Plains, New York; ^3^Department of Epidemiology, Rollins School of Public Health, Emory University, Atlanta, Georgia; ^4^Texas A&M School of Public Health, College Station, Texas.

Birth defects are a leading cause of infant mortality in the United States (*1*), accounting for approximately 20% of infant deaths. The rate of infant mortality attributable to birth defects (IMBD) in the United States in 2014 was 11.9 per 10,000 live births (*1*). Rates of IMBD differ by race/ethnicity (*2*), age group at death (*2*), and gestational age at birth (*3*). Insurance type is associated with survival among infants with congenital heart defects (CHD) (*4*). In 2003, a checkbox indicating principal payment source for delivery was added to the U.S. standard birth certificate (*5*). To assess IMBD by payment source for delivery, CDC analyzed linked U.S. birth/infant death data for 2011–2013 from states that adopted the 2003 revision of the birth certificate. The results indicated that IMBD rates for preterm (<37 weeks of gestation) and term (≥37 weeks) infants whose deliveries were covered by Medicaid were higher during the neonatal (<28 days) and postneonatal (≥28 days to <1 year) periods compared with infants whose deliveries were covered by private insurance. Similar differences in postneonatal mortality were observed for the three most common categories of birth defects listed as a cause of death: central nervous system (CNS) defects, CHD, and chromosomal abnormalities. Strategies to ensure quality of care and access to care might reduce the difference between deliveries covered by Medicaid and those covered by private insurance.

This analysis used 2011–2013 Linked Birth and Infant Death Data from the National Vital Statistics System* for infants aged <1 year born to U.S. residents. The 2003 revision of the birth certificate included information on the payment source for delivery for the first time; thus, analysis of this variable was limited to states that adopted the 2003 revision. In 2011, 33 states and the District of Columbia (DC) (representing 76% of U.S. births) used the 2003 revision; in 2012, 36 states and DC (83%) used the revision, and in 2013, 38 states and DC used the revision (86%). Approximately 1.0%–1.2% of infant death records could not be linked to their corresponding birth certificates. The linkage completion by state ranged from 95.5%–100%, with approximately 50% of states linking all of their records each year. To accommodate nonlinked death records, estimates of the number of infant deaths for each state were weighted according to the percentage of records linked to a birth certificate by state and age group at death (*6*). Gestational age was based on last menstrual period (LMP). Birth and death records with unknown gestational age, gestational age of <20 weeks or >44 weeks, and implausible combinations of gestational age and birthweight (*7*) were excluded (7.9% of infant deaths and 1.2% of live births). Births not covered by Medicaid or private insurance (18.8% of infant deaths and 17.1% of live births) were included in the totals used for rate calculations. Deaths attributable to major birth defects were defined as those for which the underlying cause of death on the death certificate was classified as a birth defect according to the *International Classification of Diseases, 10th Revision*, codes Q00.0–Q99.9. Exceptions included the following: undescended testicles (Q53.1, Q53.2, and Q53.9) or cardiovascular conditions that were not considered structural heart defects (Q27.0–Q28.9); preterm births with an underlying cause of death considered to be a complication of prematurity (i.e., lung hypoplasia [Q33.6], persistent foramen ovale [PFO, Q21.1], and patent ductus arteriosus [PDA, Q25.0]); and all neonatal deaths among term infants with PFO or PDA as the underlying cause.

Estimates for IMBD rates and 95% confidence intervals by payment source for delivery were calculated for each stratum of the key variables: maternal race/ethnicity (non-Hispanic white, non-Hispanic black, Hispanic, and other race), maternal age (<20, 20–34, and >34 years), infant gestational age (<37 and ≥37 weeks), and infant age group at death (<28 days and ≥28 days to <1 year). IMBD rates by payment source were estimated separately for the three largest defect categories listed as a cause of death (CNS defects [Q00.0–Q07.9]; CHD [Q20.0–Q26.9]; and chromosomal abnormalities [Q90.0–Q99.9]). To assess the association between payment source for delivery and IMBD, Poisson regression was used to estimate the rate ratio, adjusted for maternal age group and race/ethnicity, comparing neonatal and postneonatal IMBD among infants whose deliveries were covered by Medicaid compared with those covered by private insurance, stratified by gestational age at birth.

The linked birth/infant death records from states that adopted the 2003 revision of the birth certificate during 2011–2013 included data from 9,542,603 live births (80.6% of the U.S. total) and 53,002 infant deaths (74.5% of the total). For 11,111 (21.0%) infant deaths, a birth defect was noted as the underlying cause of death, an overall IMBD rate of 11.6 per 10,000 live births. The rate of IMBD varied by maternal race/ethnicity (non-Hispanic white, 10.9; non-Hispanic black, 13.6; and Hispanic, 12.6 per 10,000 live births) and by gestational age (preterm, 50.2; and term, 6.8 per 10,000 live births) (Table 1). Approximately 15% of all infant deaths and 70% of IMBD occurred in the neonatal period. CNS defects, CHD, and chromosomal abnormalities accounted for 57% of neonatal and 76% of postneonatal IMBD.

**TABLE 1:** Maternal and infant characteristics among cases of infant mortality attributable to birth defects (IMBD), by payment source for delivery*― United States, 2011–2013

Characteristic	Total^†^			Private insurance			Medicaid		
	No. of IMBD cases	No. of live births	Rate^§^ (95% CI)	No. of IMBD cases	No. of live births	Rate^§^ (95% CI)	No. of IMBD cases	No. of live births	Rate^§^ (95% CI)
**Total**	**11,111**	**9,542,603**	**11.6 (11.4–11.9)**	**4,227**	**4,391,048**	**9.6 (9.3–9.9)**	**5,580**	**4,163,142**	**13.4 (13.1–13.8)**
**Infant age group at death^¶^**									
Neonatal	7,767	9,542,603	8.1 (8.0–8.3)	3,020	4,391,048	6.9 (6.6–7.1)	3,787	4,163,142	9.1 (8.8–9.4)
Postneonatal	3,344	9,542,603	3.5 (3.4–3.6)	1,207	4,391,048	2.8 (2.6–2.9)	1,793	4,163,142	4.3 (4.1–4.5)
Gestational age at birth**									
Preterm	5,375	1,069,836	50.2 (48.9–51.6)	2,068	442,736	46.7 (44.7–48.7)	2,679	517,340	51.8 (49.8–53.7)
Term	5,736	8,472,767	6.8 (6.6–6.9)	2,159	3,948,312	5.5 (5.2–5.7)	2,901	3,645,802	8.0 (7.7–8.2)
**Maternal race/Ethnicity**									
White, non-Hispanic	5,548	5,090,511	10.9 (10.6–11.2)	2,901	3,039,303	9.5 (9.2–9.9)	2,082	1,617,825	12.9 (12.3–13.4)
Black, non-Hispanic	1,861	1,365,718	13.6 (13.0–14.2)	426	349,951	12.2 (11.0–13.3)	1,246	889,764	14.0 (13.2–14.8)
Hispanic	2,953	2,338,059	12.6 (12.2–13.1)	565	577,763	9.8 (9.0–10.6)	1,921	1,412,422	13.6 (13.0–14.2)
Other	582	672,661	8.7 (7.9–9.4)	269	385,008	7.0 (6.2–7.8)	256	214,677	11.9 (10.5–13.4)
**Maternal age (yrs)**									
<20	1,033	742,117	13.9 (13.1–14.8)	158	116,418	13.6 (11.5–15.7)	760	551,857	13.8 (12.8–14.8)
20–34	7,923	7,374,464	10.7 (10.5–11.0)	2,984	3,361,206	8.9 (8.6–9.2)	4,002	3,237,908	12.4 (12.0–12.7)
>34	2,155	1,426,022	15.1 (14.5–15.8)	1,085	913,424	11.9 (11.2–12.6)	818	373,377	21.9 (20.4–23.4)

* Includes residents during 2011–2013 of states that used the 2003 revised U.S. standard birth certificate, which added a checkbox indicating principal payment source for delivery: 33 states in 2011, 36 in 2012, and 38 in 2013.

^†^ Includes Medicaid, private insurance, self-pay, other, and unknown categories of payment source for delivery.

**^§^** Number of IMBD cases per 10,000 live births.

^¶^ Neonatal mortality is death of an infant with birth defects at <28 days of age. Postneonatal mortality is death of an infant with birth defects at ≥28 days to <1 year.

** Preterm birth is classified as <37 completed weeks of gestation. Term birth is classified as ≥37 completed weeks of gestation.

The IMBD rate overall was higher for deliveries covered by Medicaid (13.4 per 10,000 live births) than for deliveries covered by private insurance (9.6) (Table 1). The IMBD rate also was higher for deliveries covered by Medicaid when stratified by each of the key variables, except for maternal age <20 years (Table 1).

Payment source for delivery was associated with IMBD rates (Table 2). Among preterm births, neonatal and postneonatal IMBD rates were 12% (adjusted rate ratio [aRR] = 1.12; 95% confidence interval [CI] = 1.04–1.20) and 49% (aRR = 1.49; 95% CI = 1.29–1.72) higher, respectively, for infants whose delivery was covered by Medicaid than for those covered by private insurance. Among term births, neonatal and postneonatal IMBD rates were 44% (aRR = 1.44; CI = 1.33–1.56) and 45% (aRR = 1.45; CI = 1.31–1.60) higher, respectively, for infants whose delivery was covered by Medicaid, compared with infants whose delivery was covered by private insurance.

**TABLE 2:** Rates* of infant mortality attributable to birth defects (IMBD), by gestational age at birth,^†^ payment source for delivery,^§^ birth defect category, and infant age group at death^¶^ ― United States, 2011–2013

Birth defect category	Preterm			Term		
	Private insurance	Medicaid	Adjusted rate ratio** (95% CI)	Private insurance	Medicaid	Adjusted rate ratio** (95% CI)
**Total^††^**						
**Neonatal**	**38.2**	**39.6**	**1.12 (1.04–1.20)**	**3.3**	**4.7**	**1.44 (1.33–1.56)**
**Postneonatal**	**8.1**	**11.7**	**1.49 (1.29–1.72)**	**2.2**	**3.2**	**1.45 (1.31–1.60)**
**Central nervous system defects**						
Neonatal	5.6	6.6	1.17 (0.98–1.40)	0.6	0.8	1.37 (1.13–1.66)
Postneonatal	0.7	1.4	1.54 (0.98–2.43)	0.2	0.4	1.39 (1.03–1.89)
**Congenital heart defects**						
Neonatal	5.4	5.6	1.11 (0.92–1.34)	0.8	1.2	1.46 (1.24–1.70)
Postneonatal	3.5	4.9	1.40 (1.12–1.74)	1.1	1.6	1.43 (1.24–1.64)
**Chromosomal abnormalities**						
Neonatal	9.2	7.4	1.00 (0.85–1.16)	0.8	0.9	1.22 (1.03–1.44)
Postneonatal	1.5	2.1	1.81 (1.30–2.53)	0.4	0.6	1.48 (1.18–1.87)

* Number of IMBD cases per 10,000 live births among residents during 2011–2013 of states that used the 2003 revised birth certificate: 33 states in 2011, 36 in 2012, and 38 in 2013.

^†^ Preterm birth is classified as <37 completed weeks of gestation. Term birth is classified as ≥37 completed weeks of gestation.

^§^ Includes residents during 2011–2013 of states that used the 2003 revised U.S. standard birth certificate, which added a checkbox indicating principal payment source for delivery: 33 states in 2011, 36 in 2012, and 38 in 2013.

^¶^ Neonatal mortality is death of an infant with birth defects at <28 days of age. Postneonatal mortality is death of an infant with birth defects at ≥28 days to <1 year.

** Infant births covered by Medicaid compared with births covered by private insurance, adjusted for maternal age group and race/ethnicity.

^††^ Includes infants born to Hispanic, non-Hispanic black, non-Hispanic white women, and women of other race/ethnicity.

Among preterm births, postneonatal mortality for deliveries covered by Medicaid was 40% higher for CHD and 81% higher for chromosomal abnormalities, compared with those covered by private insurance. Among term births, neonatal mortality for deliveries covered by Medicaid was 37% higher for CNS defects, 46% higher for CHD, and 22% higher for chromosomal abnormalities, compared with those covered by private insurance. Postneonatal mortality was 39% higher for CNS defects, 43% higher for CHD, and 48% higher for chromosomal abnormalities among term deliveries covered by Medicaid than among deliveries covered by private insurance.

## Discussion

The analysis of differences in IMBD according to source of payment was possible because of the addition of a checkbox for payment source for delivery on the 2003 revision of the U.S. standard birth certificate. This variable has been assessed for data quality with moderate to substantial validity (*5*). Differences in IMBD between deliveries covered by Medicaid and those covered by private insurance were observed across categories of gestational age at birth and age group at death. Postneonatal IMBD for preterm infants, and both neonatal and postneonatal IMBD for term infants were approximately 45% higher for deliveries covered by Medicaid than those covered by private insurance. Postneonatal mortality was higher for deliveries covered by Medicaid than those covered by private insurance for the three most common categories of birth defects listed as a cause of death: CNS, CHD, and chromosomal abnormalities.

Although IMBD differed by payment source for delivery, the factors underlying this difference are not known. Differences in health status, access to and utilization of preconception health care, prenatal care, prenatal screening, and termination of pregnancies for fetal anomalies, as well as changes in insurance policies and Medicaid coverage over time could have influenced these findings (*8*,*9*).

The findings in this report are subject to at least four limitations. First, although 76%–86% of all live births were captured in this analysis, data from 12 to 17 states were not included; thus, these results are not generalizable to all states. Second, because of small numbers, it was not possible to examine other payment sources for delivery, including those deliveries that were not covered by any insurance. Third, deaths for which birth defects were listed as a contributing cause of death but not the underlying cause were not included, which likely resulted in an underestimation of IMBD. Finally, misclassification of the underlying cause of death, gestational age based on LMP (*10*), and payment source for delivery might differ by factors considered in these analyses. It is possible that mothers with high-risk pregnancies switched their payment source during pregnancy, which could have increased IMBD rates among Medicaid-covered births.

Birth defects are serious conditions that affect about one in 33 births, and in 2011–2013, one in five infant deaths had a birth defect listed as the underlying cause of death. Although IMBD rates are declining because of improvements in treatment and early detection, strategies need to be implemented to reduce IMBD for all deliveries, regardless of payment source.

**Summary**

**What is already known about this topic?**

Infant mortality attributable to birth defects (IMBD) differs by race/ethnicity, gestational age, and age group at death.

**What is added by this report?**

The rates of IMBD among preterm infants whose deliveries were covered by Medicaid were 12% and 49% higher during the neonatal and postneonatal periods, respectively, than IMBD rates among preterm infants whose deliveries were covered by private insurance. IMBD rates among term infants whose deliveries were covered by Medicaid were 44% and 45% higher during the neonatal and postneonatal periods, respectively, than rates among term infants whose deliveries were covered by private insurance.

**What are the implications for public health practice?**

Rates of infant mortality attributable to birth defects are higher for births covered by Medicaid than for those covered by private insurance. Strategies to ensure quality of care and access to care might reduce the differences between deliveries covered by Medicaid and those covered by private insurance.

## Footnotes

* https://www.cdc.gov/nchs/nvss/linked-birth.htm.
